# Reduced immunogenicity of a live *Salmonella enterica* serovar Typhimurium vaccine in aged mice

**DOI:** 10.3389/fimmu.2023.1190339

**Published:** 2023-05-03

**Authors:** Jessica C. Allen, Franklin R. Toapanta, Scott M. Baliban, Marcelo B. Sztein, Sharon M. Tennant

**Affiliations:** ^1^ Center for Vaccine Development and Global Health, University of Maryland School of Medicine, Baltimore, MD, United States; ^2^ Department of Medicine, University of Maryland School of Medicine, Baltimore, MD, United States; ^3^ Department of Pediatrics, University of Maryland School of Medicine, Baltimore, MD, United States

**Keywords:** *Salmonella*, vaccine, live-attenuated, aged, immunosenescence

## Abstract

**Introduction:**

Non-typhoidal *Salmonella* (NTS) is responsible for a high burden of foodborne infections and deaths worldwide. In the United States, NTS infections are the leading cause of hospitalizations and deaths due to foodborne illnesses, and older adults (≥65 years) are disproportionately affected by *Salmonella* infections. Due to this public health concern, we have developed a live attenuated vaccine, CVD 1926 (I77 Δ*guaBA* Δ*clpP* Δ*pipA* Δ*htrA*), against *Salmonella enterica* serovar Typhimurium, a common serovar of NTS. Little is known about the effect of age on oral vaccine responses, and due to the decline in immune function with age, it is critical to evaluate vaccine candidates in older age groups during early product development.

**Methods:**

In this study, adult (six-to-eight-week-old) and aged (18-month-old) C57BL/6 mice received two doses of CVD 1926 (10^9^ CFU/dose) or PBS perorally, and animals were evaluated for antibody and cell-mediated immune responses. A separate set of mice were immunized and then pre-treated with streptomycin and challenged orally with 10^8^ CFU of wild-type *S*. Typhimurium SL1344 at 4 weeks postimmunization.

**Results:**

Compared to PBS-immunized mice, adult mice immunized with CVD 1926 had significantly lower *S*. Typhimurium counts in the spleen, liver, and small intestine upon challenge. In contrast, there were no differences in bacterial loads in the tissues of vaccinated versus PBS aged mice. Aged mice exhibited reduced *Salmonella*-specific antibody titers in the serum and feces following immunization with CVD 1926 compared to adult mice. In terms of T cell responses (T-CMI), immunized adult mice showed an increase in the frequency of IFN-γ- and IL-2-producing splenic CD4 T cells, IFN-γ- and TNF-α-producing Peyer’s Patch (PP)-derived CD4 T cells, and IFN-γ- and TNF-α-producing splenic CD8 T cells compared to adult mice administered PBS. In contrast, in aged mice, T-CMI responses were similar in vaccinated versus PBS mice. CVD 1926 elicited significantly more PP-derived multifunctional T cells in adult compared to aged mice.

**Conclusion:**

These data suggest that our candidate live attenuated *S.* Typhimurium vaccine, CVD 1926, may not be sufficiently protective or immunogenic in older humans and that mucosal responses to live-attenuated vaccines decrease with increasing age.

## Introduction

Non-typhoidal *Salmonella* (NTS) is a major cause of morbidity and mortality worldwide. The global burden of NTS disease is estimated to be 93 million diarrheal cases and 150,000 deaths each year (1). Specifically in the United States, NTS is the leading cause of hospitalizations and deaths due to foodborne infections (2). During 2021, the Foodborne Diseases Active Surveillance Network (FoodNet) reported 7,148 *Salmonella* laboratory-diagnosed infections, 1,974 hospitalizations and 52 deaths among 10 U.S. states (2). Alarmingly, 5,620 of the global deaths caused by infection with *Salmonella* spp. in 2019 were attributable to antimicrobial resistant (AMR) strains; AMR infections are associated with worse clinical outcomes (3, 4). The Centers for Disease Control and Prevention lists NTS as a serious antibiotic resistant threat that requires prompt and sustained action (5).

Most people generally experience self-limiting NTS gastroenteritis lasting 5-7 days. However, those who are malnourished, HIV-positive, infants, or older (≥65 years) are at increased risk for invasive infections that can result in bacteremia, meningitis, and/or death (6, 7). In the United States, rates of severe illness and hospitalization due to NTS have been demonstrated to be highest in individuals ≥65 years of age and increase with age (8–10). Individuals ≥65 years have a ten-fold higher case fatality rate than those aged 5-64 years (3.0% versus 0.3%) (10, 11). Older adults with gastrointestinal illness are more likely to experience extraintestinal manifestations such as septicemia, meningitis, acute renal failure, hemolytic uremic syndrome and arrhythmias, thereby resulting in further health complications for these individuals (10, 12). This poses major risks for long-term care facilities where many older individuals share living spaces and are prone to transmitting NTS to one another (12, 13).

NTS vaccines could be used to protect older adults but currently there are no licensed NTS vaccines available. We have developed a live attenuated *S*. Typhimurium vaccine candidate, CVD 1926 (I77 Δ*guaBA* Δ*clpP* Δ*pipA* Δ*htrA*), against *Salmonella enterica* serovar Typhimurium (14, 15), one of the most common serotypes of NTS worldwide (16–18). The *guaBA* deletion is the primary attenuating mutation which produces an auxotrophic *S*. Typhimurium strain that requires guanine for growth (19). The *clpP* mutation further attenuates the bacteria and produces hyperflagellation. Deletion of *pipA* and *htrA* augments vaccine tolerability (15). In our previous work, we have used non-human primate and murine models to evaluate CVD 1926 (14, 15). We showed that CVD 1926 was able to protect rhesus macaques against moderate-to-severe diarrhea with a vaccine efficacy of 80%; vaccinated animals experienced diarrhea for fewer days and had significantly lower organ burden levels compared to unvaccinated animals following challenge with wild-type *S*. Typhimurium (15). In mice, CVD 1926 was well-tolerated and immunogenic (100% seroconversion of anti-lipopolysaccharide [LPS] serum IgG) in young adult BALB/c mice and protected them against lethal challenge with wild-type (WT) *S.* Typhimurium at 1 and 3 months postimmunization (14). These results indicate that CVD 1926 is a promising vaccine candidate in adult animals. However, this and other live NTS vaccines have not yet been evaluated in aged animal models.

Although vaccination is the most effective intervention in preventing infections, older adults experience reduced vaccine efficacy and/or immunogenicity (e.g., to vaccines targeting SARS-CoV-2, influenza virus, *Streptococcus pneumoniae*, and herpes zoster [shingles]) (20–27) due to a decline and/or dysregulation in immunity with age, termed immunosenescence. However, our current understanding of vaccine responses in older age groups is primarily derived from studies using parenteral vaccines, and little is known about peroral vaccine responses in older adults. To our knowledge, only two oral live attenuated vaccines have been administered to the elderly; a typhoid vaccine (Ty21a) and a pentavalent rotavirus vaccine (RotaTeq^®^) (28, 29). After Ty21a immunization, the terminal ileum resident T cells of elderly volunteers showed reduced IL-17A and IL-2 production, compared to younger adults; indicating that specific mucosal immune responses were weaker in older volunteers compared to adults. As for RotaTeq^®^, this vaccine was safe and well tolerated in older adults. After one dose, serum neutralizing antibodies against rotavirus were elevated in aged volunteers. However, a younger adult comparator was not included in this study; thus, the effect of age on RotaTeq^®^ vaccine responses remain unknown. In mice, Fujihashi et al. reported that mucosal immunosenescence to soluble protein antigens can occur as early as 1 year of age; 12- to 14-month-old mice failed to generate fecal IgA responses to oral immunization with ovalbumin adjuvanted with cholera toxin (30, 31). This was associated with fewer antibody-forming cells in the gut-associated lymphoid tissue, compared to adult mice (30, 32). Therefore, there is some evidence that mucosal immunosenescence progresses with age, but it is unknown whether vaccination with live oral NTS vaccines will be less protective and immunogenic in older individuals.

The goal of the current study was to evaluate the immunogenicity of a live attenuated *S*. Typhimurium oral vaccine candidate, CVD 1926, in the context of immunosenescence by measuring mucosal and systemic vaccine responses in 18-month-old and 6- to 8-week-old mice. To this end, we assessed the following parameters: (i) protection of vaccinated mice against extraintestinal infection with *S*. Typhimurium, (ii) humoral immunity by measuring antigen-specific serum IgG and fecal IgA levels, and (iii) T cell mediated immunity (T-CMI) by measuring IFN-γ, TNF-α, and IL-2 production and/or CD107a expression in mucosal and splenic CD4 and CD8 T cells. These findings will expand our knowledge of age-associated deficits in generating local and systemic immunity to live *S*. Typhimurium vaccines and shed light on oral vaccine responses within older hosts.

## Materials and methods

### Animals and ethics statement

All animal studies were performed in facilities that are accredited by the Association for Assessment and Accreditation of Laboratory Animal Care. Mice were housed under specific pathogen–free conditions at the University of Maryland School of Medicine, and all the procedures were approved by the University of Maryland School of Medicine Institutional Animal Care and Use Committee (protocol no. 0619004). C57BL/6 mice (both sexes) were used to examine T-CMI, antibody responses and to assess protection against bacterial burden using a streptomycin mouse model. Six- to 8-week-old (adult) C57BL/6 mice were purchased from The Jackson Laboratory (Bar Harbor, ME). Eighteen-month-old (aged) C57BL/6 mice were acquired from the National Institute on Aging aged rodent colony or purchased from The Jackson Laboratory. BALB/c mice (female only) were used to examine antibody responses and to determine protection against lethal challenge. Six- to 8-week-old BALB/c mice were purchased from Charles River Laboratories (Wilmington, MA). For 18-month-old BALB/c mice, 6- to 8-week-old mice were purchased from Charles River Laboratories and housed at the University of Maryland School of Medicine until 18 months of age.

### Bacterial strains, medium, and culture conditions

Bacterial strains used in this study are shown in **Table 1**. All bacterial strains were maintained in animal-product-free Hy-Soy (HS) medium (10 g/L Soytone [Teknova, Hollister, CA], 5 g/L Hy-yest [Kerry Bio-Science, Beloit, WI] and 5 g/L sodium chloride [American Bio, Natick, MA]) at 37°C. When needed, agar (Sigma–Aldrich, St. Louis, MO) was added at 15 g/L. For CVD 1926, medium was supplemented with guanine (0.005% weight/volume [w/v] final concentration; Sigma–Aldrich). For *S*. Typhimurium SL1344, medium was supplemented with 50 μg/mL streptomycin sulfate (Research Products International, Mt. Prospect, IL).

**Table 1:** *S*. Typhimurium strains used in this study.

Strain	Characteristics and purpose	Reference
SL1344	Streptomycin-resistant, ST19; used to challenge C57BL/6 mice using the streptomycin mouse model	(33, 34)
I77	Clinical blood isolate from Mali, antibiotic sensitive, ST19; used to prepare lysate for cell stimulation and to challenge BALB/c mice to determine protection against lethal challenge	(35)
CVD 1926	*S*. Typhimurium I77 Δ*guaBA* Δ*clpP* Δ*pipA* Δ*htrA* vaccine strain; used to immunize C57BL/6 and BALB/c mice	(14)
CVD 1925	Reagent strain for FliC isolation	(19)
CVD 1925 (pSEC10-*wzzB*)	Reagent strain for COPS isolation	(36)

### Immunization and challenge

*Salmonella* strains were streaked onto HS agar (+ 0.005% guanine for CVD 1926) and incubated at 37°C for 18-20 h. Bacteria were then resuspended in sterile PBS and washed twice by centrifugation at 4°C. Bacteria were concentrated by centrifugation or diluted with sterile PBS to achieve the correct dosage. Adult and aged C57BL/6 mice were immunized by peroral gavage with either 10^9^ colony forming units (CFU) of CVD 1926 suspended in 100 μL PBS or 100 μL PBS alone on days 0 and 28. Adult and aged BALB/c mice received either 10^9^ CFU of CVD 1926 suspended in 100 μL PBS or 100 μL PBS by oral gavage on days 0, 21, and 42. Immunized mice were monitored for adverse effects following immunization including lethargy, difficulty breathing, inability to ambulate, and weight loss. Blood was collected one day prior to each immunization or challenge to determine serum antibody titers. Fecal pellets were collected at day -1 and day 55 (for C57BL/6 mice) or 69 (for BALB/c mice) to determine fecal antibody titers.

Immunized C57BL/6 mice were challenged using the streptomycin mouse model (33, 37). Briefly, 4 weeks after the last immunization, mice were fasted for 4 hours before being administered 20 mg streptomycin suspended in 100 µL sterile water by peroral gavage. Food and water were returned immediately. Twenty hours later, food and water were removed for 4 hours, and then mice were infected by peroral gavage with 10^8^ CFU/100 µL of the streptomycin-resistant strain *S*. Typhimurium SL1344 suspended in PBS. On day 3 post-infection, mice were euthanized, and the spleen, liver, and gastrointestinal tract were harvested, homogenized, and diluted to a concentration of 100 mg tissue/mL PBS. Bacterial counts were determined by spread plating on HS agar containing 50 μg/mL streptomycin. Data are presented as CFU/organ which represents the total *S*. Typhimurium SL1344 recovered from the whole organ.

Immunized BALB/c mice were challenged with a lethal dose of wild-type *S*. Typhimurium I77 to determine vaccine efficacy. Briefly, immunized mice were challenged perorally with 100 x LD_50_ (3 x 10^6^ CFU/100 µL) wild-type *S*. Typhimurium I77 four weeks post-immunization (day 70). Mice were monitored for up to 30 days for weight loss and signs of illness and euthanized if they met alternative endpoint criteria (e.g., lost ≥20% of their starting body weight) and scored as having succumbed to infection.

### Sample collection and assessment of antibody responses

Venous blood was collected and serum separated using serum gel tubes (Sarstedt, Numbrecht, Germany). Serum was isolated after centrifugation at 10,000 *x g* for 5 min. Fecal pellets were weighed and suspended in ice-cold PBS containing 0.01% sodium azide and 1% protease inhibitor cocktail at a concentration of 100 mg stool/mL (MilliporeSigma, St. Louis, MO). Debris were removed by centrifugation at 15,000 *x g* for 10 min at 4°C, and the fecal supernatants were collected. All samples were stored at -80°C until analysis.

Vaccine-induced antibody responses were measured by enzyme-linked immunosorbent assay (ELISA). Briefly, 96-well medium binding plates (Greiner Bio-One, Monroe, NC) were coated with either *S*. Typhimurium core-and O-polysaccharide (COPS) or FliC antigens (19, 36) in PBS at a concentration of 5 μg/mL and incubated overnight at 4°C. Plates were washed with PBS-T (PBS containing 0.05% Tween 20) and blocked with PBS + 10% Omniblok non-fat, dry milk for 2 h at 37°C. Samples were serially diluted in PBS-T + 10% Omniblok, transferred to blocked ELISA plates, and incubated for 1 h at 37°C. Plates were washed and incubated for 1 h at 37°C with horseradish peroxidase (HRP)-labeled anti-mouse IgG (KPL, Gaithersburg, MD) or HRP-labeled anti-mouse IgA (KPL). After washing, substrate (3,3’,5,5’-tetramethylbenzidine; KPL) was added, and the plates were incubated for 10 min in darkness. The reaction was stopped with the addition of 1 M H_3_PO_4_, and the absorbance at 450 nm was recorded using a VersaMax microplate reader (Molecular Devices, San Jose, CA). ELISA titers were calculated by interpolation of absorbance values on a standard curve. The endpoint titers reported as ELISA units (EU)/mL represent the inverse of the serum dilution that produced an absorbance value of 0.2 above the blank. Seroconversion in vaccinated mice was defined as a 4-fold increase in the antibody titer compared to the pre-immunization titer.

### Isolation of mononuclear cells from the spleen and gut

Single cell suspensions of the spleen and Peyer’s Patches were made by mechanical dissociation of organs through 70-µm-pore size nylon filters. Red blood cells in the spleen were lysed with Ammonium-Chloride-Potassium (ACK) lysis buffer (Gibco, Grand Island, NY). Cells were washed with PBS and then resuspended in complete RPMI 1640 medium (Gibco) supplemented with 10% fetal bovine serum (FBS; Gemini Bioproducts, West Sacramento, CA), 2 mM L-glutamine (Gibco), 1X non-essential amino acids (Gibco), 10 mM HEPES (Gibco), 2.5 mM Sodium pyruvate (Gibco), 100 U/mL penicillin (Sigma-Aldrich), 100 µg/mL streptomycin (Sigma-Aldrich), and 50 μg/mL gentamicin (Gibco).

### Preparation of *S*. Typhimurium lysate

*S*. Typhimurium I77 was grown on HS agar for 18-20 h at 37°C. Bacteria were harvested, washed in sterile PBS at 4°C and heat-inactivated at 65°C for 30 minutes. Suspensions were then sonicated using Fisherbrand™ Model 120 Sonic Dismembrator (Fisher Scientific, Waltham, MA) for 10 short bursts of 10 seconds at 20 Khz frequency. After sonication, suspensions were centrifuged at 2800 *x g* for 15 minutes and the supernatant was collected for protein quantification. To determine the protein concentration of the lysate, the Pierce™ BCA Protein Assay Kit was used (ThermoFisher, Waltham, MA).

### *Ex-vivo* stimulation and flow cytometry staining

One million cells from the spleen or Peyer’s Patches were stimulated with either (i) media, (ii) phorbol 12-myristate 13-acetate (PMA; 20 ng/µL) and ionomycin (1 µg/mL), or (iii) *S.* Typhimurium I77 cell lysate (5 μg/mL) for 16-18 h in round-bottom tubes (Corning, Kennebunk, ME). Cells were then incubated for 4 h in the presence of anti-CD107a-BV510™ (clone 1D4B; Biolegend, San Diego, CA) before overnight incubation with protein transport blockers monensin (1 μg/mL; Sigma) and brefeldin A (2 μg/mL; Biolegend). The next day, cells were transferred to V-shaped 96-well plates (Corning) and stained for flow cytometry. Briefly, 1 x 10^6^ cells were added to a well, washed with PBS, and stained for viability using LIVE/DEAD™ Fixable Yellow stain (Invitrogen, Thermo Fisher Scientific). For cell-surface staining, cells were blocked with anti-mouse CD16/32 (clone 93, Biolegend) for 10 minutes and stained extracellularly with an antibody cocktail (**Table S1**) for 30 minutes on ice. Pacific Orange-Streptavidin (Invitrogen) was added for 30 minutes on ice as a secondary staining step. Cells were then permeablized and fixed using the eBioscience™ Intracellular Fixation & Permeablization Buffer Set (Invitrogen) according to the manufacturer’s instructions. An antibody cocktail against intracellular targets (**Table S1**) was added to cells for 30 minutes at room temperature. Cells were then washed with FACS buffer and fixed with 2% paraformaldehyde (PFA) in PBS. Samples were acquired using a Cytek^®^ Aurora spectral flow cytometer and analyzed using SpectroFlo^®^ Software (Cytek^®^ Biosciences, Fremont, CA). T cell responses against *S.* Typhimurium I77 were expressed as a net percentage of cytokine or CD107a positive cells (i.e., percentage of *S.* Typhimurium-stimulated cells minus percentage of cells cultured with media only). For each cytokine and CD107a, a cutoff to determine “true responders” was included, as described (15). The cutoff point was the highest value for the cytokine/CD107a identified in the PBS group post-vaccination. Any sample with a net percentage higher than the cutoff was considered a true responder.

### Statistical analysis

Data were analyzed using GraphPad Prism 7 Software (La Jolla, CA, USA). A *p*-value equal to or below 0.05 was considered significant for each test. For ELISA and T cell analyses, statistical comparisons were accomplished using a Mann-Whitney U test (two-tailed, α = 0.05). For assessing vaccine efficacy, seroconversion and cytokine responders, Fisher’s exact test was used. Correlations between bacterial organ burden and α-COPS IgG titers were calculated using a Spearman correlation test (two-tailed, α = 0.05). Survival curves of challenged mice were compared by the log-rank test. Vaccine efficacy (VE) was calculated based on the attack rate (AR) in control and vaccinated mice as follows: VE = (AR_controls_ - AR_vaccinated_)/AR_controls_) × 100.

## Results

### CVD 1926 elicits poor protection against challenge with *S*. Typhimurium in aged versus adult C57BL/6 mice

**Figure 1:**
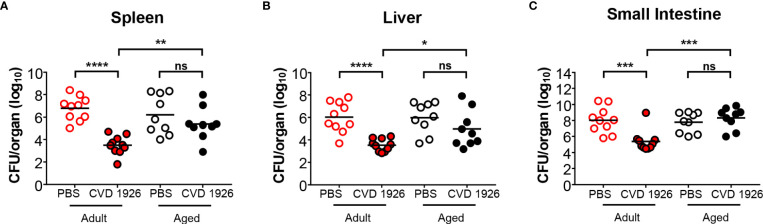
Bacterial burden of adult and aged C57BL/6 mice after challenge with wild-type *S.* Typhimurium. Bacterial burden in the **(A)** spleen, **(B)** liver, and **(C)** small intestine of adult and aged mice (n=5 of each sex) immunized with either PBS or CVD 1926, and then subsequently challenged perorally with 10^8^ CFU of *S*. Typhimurium SL1344. Twenty-four hours prior to challenge, mice were pretreated with peroral streptomycin. Median represented by bar (ns, not significant; *p ≤ 0.05; **p ≤ 0.01 ***p ≤ 0.001; ****p ≤ 0.0001 by Mann-Whitney).

We found that CVD 1926 was safe, and no adverse effects were observed following immunization of 6- to 8-week-old (adult) or 18-month-old (aged) C57BL/6 mice (n=10). We subsequently used the streptomycin challenge model to evaluate protection against colonization with *S*. Typhimurium (33, 37). At day 3 after challenge, the spleen, liver, and small intestine of CVD 1926-immunized adult mice contained significantly less *S*. Typhimurium, ~3 log_10_ CFU lower, compared to the bacterial load of PBS mice (**Figure 1**). In contrast, CVD 1926 failed to confer protection in aged mice, as there were no significant differences in *S*. Typhimurium levels within the spleen, liver, and small intestine between vaccinated and unvaccinated animals (**Figure 1**). Further, for CVD 1926-immunized animals, the adult mice showed significantly lower organ burden in the spleen, liver, and small intestine compared to aged mice (spleen: *p* = 0.003; liver: *p* = 0.02; small intestine *p* = 0.0004). To determine if this phenotype is consistent in a different mouse strain and using a lethal infection model, we assessed vaccine efficacy in BALB/c mice. Three immunizations were used here to compare results to previous findings using this model (14). We observed a vaccine efficacy of 64% (*p* = 0.0007) against lethal challenge in adult BALB/c mice, but only 33% vaccine efficacy (*p* = 0.093) in aged BALB/c mice (**Table S2**). However, by log-rank test, there was a significant difference in survival curves between CVD 1926- and PBS-immunized animals for both the adult and aged mice (**Figure S1**).

### Aged C57BL/6 mice fail to produce robust antibody responses following immunization with CVD 1926

**Figure 2:**
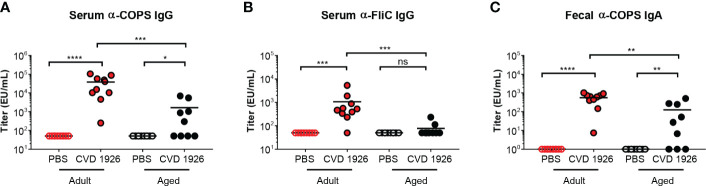
Serum IgG and fecal IgA responses from adult and aged C57BL/6 mice immunized with CVD 1926. **(A)** Anti-COPS and **(B)** anti-FliC serum IgG responses and **(C)** anti-COPS fecal IgA responses after 2 doses of PBS or CVD 1926 (day 55). Geometric mean titer represented by bar (ns, not significant; *p ≤ 0.05; **p ≤ 0.01; ***p ≤ 0.001; ****p ≤ 0.0001 by Mann-Whitney).

CVD 1926 elicited robust anti-COPS and anti-FliC serum IgG antibody titers in adult compared with aged C57BL/6 mice (anti-COPS: *p* = 0.0009; anti-FliC: *p* = 0.0003) (**Figures 2A, B**). Anti-COPS and anti-FliC serum titers in CVD 1926-immunized adult mice were significantly higher than unvaccinated mice at day 55 (anti-COPS: *p* < 0.0001; anti-FliC: *p* = 0.0001). Although aged mice vaccinated with CVD 1926 elicited significantly higher anti-COPS serum IgG titers compared to unvaccinated mice (*p* = 0.011), there was no difference in anti-FliC serum IgG titers (**Figures 2A, B**). The majority of vaccinated adult mice seroconverted to serum anti-COPS IgG (100%) and anti-FliC serum IgG (90%); however, seroconversion in the aged cohort was significantly diminished in comparison, with 56% of mice seroconverting for anti-COPS (*p* = 0.033) and 11% (*p* = 0.001) for anti-FliC serum IgG titers (**Table 2**). One day prior to challenge, fecal samples from adult mice contained significantly higher anti-COPS IgA levels compared to aged mice (*p* = 0.003) although there was no significant difference between groups in terms of seroconversion (**Figure 2C** and **Table 2**).

**Table 2:** Proportion of mice that show seroconversion following immunization with PBS or CVD 1926.

No. of mice that seroconverted/No. of mice tested (%)				
	Immunization	α-COPS IgG	α-FliC IgG	α-COPS IgA
**Adult**	PBS	0/10 (0%)	0/10 (0%)	0/10 (0%)
	CVD 1926	10/10 (100%)*	9/10 (90%)***	10/10 (100%)
**Aged**	PBS	0/9 (0%)	(0/9) (0%)	0/9 (0%)
	CVD 1926	5/9 (56%)	1/9 (11%)	6/9 (67%)

*, immunization with CVD 1926, adult versus aged; p ≤ 0.05 by Fisher’s exact test.

***, immunization with CVD 1926, adult versus aged; p ≤ 0.001 by Fisher’s exact test.

Both adult and aged BALB/c mice showed a dose-response increase in anti-COPS serum IgG levels post-vaccination, in comparison to PBS-immunized mice (**Figures S2A, C**). Although both adult and aged vaccinated mice produced high fecal IgA titers by day 69, adult mice produced significantly higher anti-COPS IgA levels than aged mice with a geometric mean titer (GMT) of 1,122.33 and 180.34, respectively (*p* < 0.0001) (**Figures S2B, D**). We investigated the relationship between anti-COPS antibody levels and *S*. Typhimurium organ burden following challenge in adult and aged C57BL/6 mice. Anti-COPS serum IgG levels and *S*. Typhimurium CFU from the spleens of adult and aged mice were negatively correlated (r = -0.519, *p* = 0.027) (**Figure S3A**). For the liver and small intestine, there was a moderate negative association between anti-COPS serum IgG levels and organ burden but the *p*-values were not significant (liver: r = -0.455, *p* = 0.058; small intestine: r = -0.450, *p* = 0.061) (**Figures S3B, C**). Anti-COPS fecal IgA levels and CFU counts in the spleen (r = -0.479, *p* = 0.045) and liver (r = -0.584, *p* = 0.011) were negatively correlated as well (**Figures S4A, B**). Likewise, there was a negative but non-significant association between the fecal IgA titers and bacteria present within the small intestine (r = -0.461, *p* = 0.054) (**Figure S4C**).

### Reduced T-CMI responses in aged C57BL/6 mice receiving CVD 1926 compared to C57BL/6 adult mice

**Figure 3:**
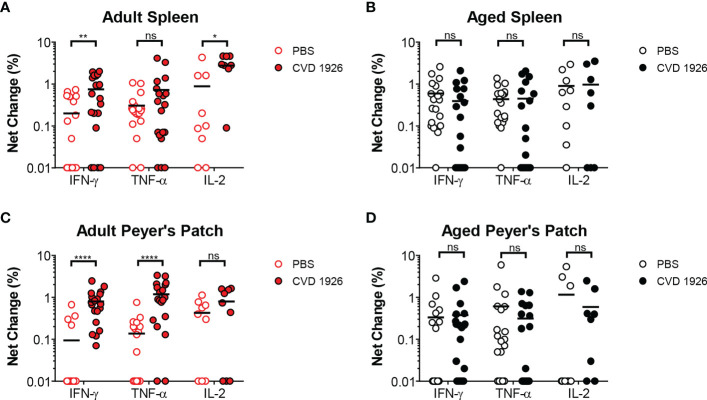
Evaluation of CD4 T cell responses elicited by CVD 1926 in C57BL/6 mice. Fourteen days following the second immunization with CVD 1926, cytokine production from CD4 T cells were assessed in the spleens **(A, B)** and Peyer’s Patches (PP; **C, D**) of adult **(A, C)** and aged **(B, D)** mice. Changes in these markers were assessed upon *ex-vivo* stimulation with *S.* Typhimurium lysate. Each point represents data from one mouse. Median represented by bar (ns, not significant; *p ≤ 0.05; **p ≤ 0.01; ****p ≤ 0.0001 by Mann-Whitney).

**Figure 4:**
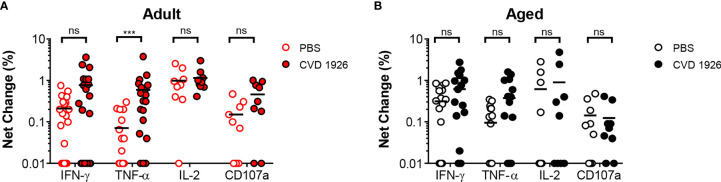
Evaluation of CD8 T cell responses elicited by CVD 1926 in C57BL/6 mice. Fourteen days following the second immunization with CVD 1926, cytokine production and CD107a upregulation from CD8 T cells were assessed in the spleens of **(A)** adult and **(B)** aged mice. Changes in these markers were assessed upon *ex-vivo* stimulation with *S.* Typhimurium lysate. Each point represents data from one mouse. Median represented by bar (ns, not significant; ***p ≤ 0.001 by Mann-Whitney).

For evaluation of systemic and mucosal T-CMI to CVD 1926 in C57BL/6 mice, splenic and Peyer’s Patch (PP)-derived cells were stimulated *ex-vivo* with wild-type *S*. Typhimurium lysate 10 days following the second immunization, and cytokine production (IFN-γ, TNF-α, IL-2) and CD107a upregulation (a T cell degranulation marker) by CD4 and CD8 T cells were assessed by intracellular flow cytometry (**Figure S5**). There were no differences in the frequencies of CD4 and CD8 T cells within the spleen and PPs of adult and aged mice (**Figure S6**). However, within the CD4 and CD8 T cell compartments, naïve aged mice had significantly more T cells expressing CD44 (**Figure S7**). *S*. Typhimurium-specific T-CMI was recorded in the spleens of adult mice as shown by higher levels of IFN-γ+ and IL-2+ CD4 T cells in the spleens of immunized adult mice compared to PBS-immunized mice (**Figure 3A**). Likewise, frequencies of antigen-specific IFN-γ+ and TNF-α+ PP-derived CD4 T cells were significantly higher in CVD 1926-immunized mice compared to unvaccinated mice (**Figure 3C**). Importantly, CVD 1926 did not elicit *S*. Typhimurium-specific T-CMI in aged mice, as there were no differences in CD4 T cell cytokine production between vaccinated and unvaccinated aged mice in the spleen or PP (**Figures 3B, D** respectively). Splenic CD8 T cells were also evaluated, but there were too few CD8 T cells in the PP to evaluate statistical differences between individual mice. In the CD8 compartment, CVD 1926-immunized adult mice demonstrated higher frequencies of TNF-α+ CD8 T spleen cells compared to non-immunized animals (**Figure 4A**). CVD 1926 did not elicit strong CD8 T-CMI in aged mice (**Figure 4B**). The proportion of CD4 and CD8 T cell responders is shown in **Table 3**.

**Table 3:** Proportion of mice that show T cell responses to CVD 1926 immunization.

	No. of mice that show T cell responses/No. of mice tested (%)									
	Splenic CD4 T cells			PP-derived CD4 T cells			Splenic CD8 T cells			
	IFN-γ	TNF-α	IL-2	IFN-γ	TNF-α	IL-2	IFN-γ	TNF-α	IL-2	CD107a
**Adult**	10/19 (53%)*	7/19 (37%)	7/9 (78%)	14/19 (74%)*	15/19 (79%)*	4/10 (40%)	8/19 (42%)	11/19 (58%)	1/10 (10%)	3/10 (30%)
**Aged**	2/19 (11%)	4/19 (21%)	2/9 (22%)	3/19 (16%)	4/19 (21%)	0/10 (0%)	5/19 (26%)	8/19 (42%)	2/10 (20%)	0/10 (0%)

*, adult versus aged; p ≤ 0.05 by Fisher’s exact test.

**Figure 5:**
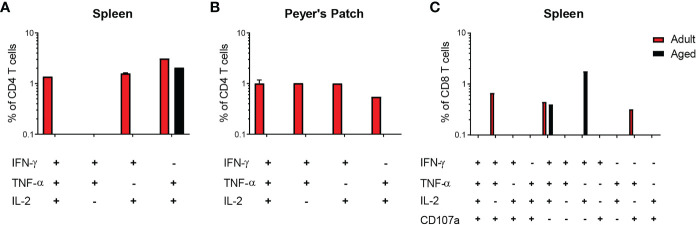
Cytokine profiles of multifunctional (MF) CD4 and CD8 T cells elicited by CVD 1926 in C57BL/6 mice. Induction of MF CD4 and CD8 T cells following immunization with CVD 1926 in vaccine responders. Fourteen days following the second immunization with CVD 1926, the ability of CD4 T cells in the **(A)** spleens and **(B)** Peyer’s Patches as well as **(C)** CD8 T cells in the spleen to produce more than one cytokine and/or CD107a expression was assessed. Only data from animals demonstrating multifunctionality are shown.

T cells capable of upregulating at least two cytokines/CD107a expression (termed multifunctional [MF]) were assessed in a subset of mice in response to *S*. Typhimurium stimulation. Amongst adult vaccine recipients, 44% demonstrated MF responses in splenic CD4 T cells, 66% in PP-derived CD4 T cells, and 33% in splenic CD8 T cells (**Table 4**). In contrast, single functionality (or no functionality) predominated in aged mice; 11% of splenic CD4 T cell responders, 0% of PP-derived CD4 T cell responders, and 22% of splenic CD8 T cell responders were MF. The number of CVD 1926-immunized animals demonstrating multifunctionality in the PP was significantly greater for adult mice than aged mice (*p* = 0.009) (**Table 4**). For MF splenic CD4 T cells from adult mice, IL-2 production was detected from all animals displaying multifunctionality (**Figure 5A**). No dominant cytokine expression profile was observed in PP-derived MF CD4 T cells since MF cells producing different combinations of IFN-γ, TNF-α and/or IL-2 were all detected (**Figure 5B**). For multifunctionality in the CD8 T cell compartment, 3/9 (33%) adult mice and 2/9 (22%) aged mice demonstrated detectable MF CD8 T cells (**Table 4**). TNF-α production (with IFN-γ, IL-2 and/or CD107) was dominant in MF CD8 T cells from adult mice, whilst IFN-γ and IL-2 production was consistent among MF CD8 T cells from aged mice (**Figure 5C**).

**Table 4:** Proportion of mice that show multifunctional (MF) T cell responses to CVD 1926 immunization.

	No. of mice that show MF T cell responses/No. of mice tested (%)		
	Splenic CD4 T cells	PP-derived CD4 T cells	Splenic CD8 T cells
**Adult**	4/9 (44%)	6/9 (66%)*	3/9 (33%)
**Aged**	1/9 (11%)	0/9 (0%)	2/9 (22%)

*, adult versus aged; p ≤ 0.05 by Fisher’s exact test.

## Discussion

Development of an effective NTS vaccine for high-risk target groups, including the elderly, remains a public health priority. In this study, we explored the impact of age-related immunosenescence on the protective ability of CVD 1926, a live-attenuated vaccine candidate for *S*. Typhimurium. We demonstrated that aged mice immunized with CVD 1926 failed to reduce bacterial burden upon challenge with wild-type *S*. Typhimurium, which was associated with lower vaccine-induced antibody titers and weaker T cell responses. Our data suggest that CVD 1926 is poorly immunogenic and less efficacious in this age group.

Aged mice immunized with CVD 1926 were unable to lower the bacterial load upon challenge which is consistent with other studies showing that aged mice failed to clear influenza, SARS-CoV-2 or *Streptococcus pneumoniae* infection after immunization (38–40). Moreover, we showed that aged mice produced poor antigen-specific antibody and T cell responses following vaccination, which are both involved in protective immunity to NTS infection in susceptible mouse strains (41); hence, consistent with the inability to control the bacterial burden.

Decreased antigen-specific antibody levels have been implicated in weak vaccine responses in older individuals (27, 42). In line with these observations, we found that aged mice produced significantly lower anti-COPS and anti-FliC serum IgG responses compared to adult mice. Notably, CVD 1926 did not elicit an anti-FliC IgG response altogether in aged mice, as evidenced by comparable titers between CVD 1926- and PBS-immunized mice. Antibodies directed at the O-antigen of LPS and flagellin protein of *Salmonella* play a critical role in mediating protection in mice and humans by promoting phagocytic uptake and/or lysing bacteria directly via complement fixation (43–46). Since prevention of bacterial dissemination relies on robust preexisting antibody responses, the inability of CVD 1926-immunzed aged mice to clear bacteria upon infection may be explained by modest levels of anti-COPS and anti-FliC serum IgG. Indeed, we observed a significant relationship between *S*. Typhimurium CFUs in the spleen and anti-COPS serum IgG levels, which is consistent with reports from sub-Saharan Africa where *Salmonella*-specific antibody titers were shown to be associated with decreased disease in humans (47, 48).

Aged C57BL/6 mice produced lower levels of anti-COPS fecal IgA with only 67% of mice seroconverting, as compared to 100% of adult mice. Similarly, fecal IgA levels from aged BALB/c mice were lower compared to adult mice post-immunization. Secretory IgA (SIgA) is proposed to entrap antigens and pathogens within the intestinal lumen, which restricts colonization of the gut mucosa, as well as invasion to extraintestinal tissues (49). Despite comparable anti-COPS serum IgG titers between adult and aged BALB/c mice post-immunization, aged BALB/c mice achieved a vaccine efficacy of only 33% (versus 64% in adult mice), suggesting a role for mucosal IgA in protection against lethality. Richards et al. demonstrated that a human SIgA monoclonal antibody against *S*. Typhimurium LPS, Sal4, limited *S*. Typhimurium invasion of Peyer’s Patches in BALB/c mice (50). Lastly, in humans, the quantity and avidity of IgA specific for *S*. Typhi Vi polysaccharide was correlated with protection against typhoid fever, thereby highlighting the importance of IgA mediated immunity (51, 52). With these studies and our finding that IgA titers in the stool negatively correlate with *S*. Typhimurium burden in the spleen and liver, we postulate that, in addition to serum IgG, antigen-specific IgA in the intestinal lumen assists in preventing bacterial dissemination to deep tissues upon oral infection. In line with this hypothesis, the age-associated decrease in fecal IgA titers likely contributes to the high bacterial burden in aged C57BL/6 mice after challenge.

CVD 1926 elicited fewer T cells capable of producing pro-inflammatory cytokines in aged mice compared to young animals. The number of T cell responders, the magnitude of cytokine production, and number of animals with MF T cells was weak in aged mice, suggesting that, collectively, CVD 1926 failed to elicit a robust T-CMI in aged mice. This is consistent with data from older adult humans that received influenza and SARS-CoV-2 mRNA vaccines and may explain the decreased protection in older animals (53). Compared to PBS-treated adult mice, CVD 1926-immunized animals showed an increase in the frequency of splenic IFN-γ and IL-2 producing CD4 T cells and IFN-γ and TNF-α producing PP-derived CD4 T cells following *in vitro* stimulation with *S*. Typhimurium lysate. Since cytokine production upon re-stimulation is a known property of effector T cells and *Salmonella* clearance requires a robust Th1 response, we postulate that the T cells of vaccinated adult mice secrete pro-inflammatory cytokines to promote bacterial killing by immune inflammatory cells in the gut, limiting extra-intestinal spread during infection (54–57). This is supported by a study showing that convalescent typhoid fever patients produced higher levels of IFN-γ, TNF-α and MIP-1β (58). Moreover, extensive studies using specimens from participants exposed to wild-type *S.* Typhi showed striking associations in the specific production of IFN-γ, TNF-α, IL-2, and other cytokines, as well as the expression of CD107, and protection from disease (59–61). MF T cells are an indicator of efficient effector responses and there is evidence that secretion of multiple cytokines synergistically support phagocytic killing (62, 63). CVD 1926 elicited multifunctionality in 44%, 66% and 33% of adult mice for splenic CD4, PP-derived CD4, and splenic CD8 T cell responses, respectively. In contrast, only 11%, 0%, and 22% of aged mice showed multifunctional cytokine production for splenic CD4, PP-derived CD4, and splenic CD8 T cell compartments, respectively. IL-2 is essential for T cell survival and its production was detected alongside IFN-γ or TNF-α in all splenic and most PP-derived CD4 MF T cells in adult mice (64). Therefore, the presence of IL-2-secreting T cells in the spleen and PP of adult mice may contribute to the robust T cell responses observed in adult but not aged mice. In addition to functional defects, T cells experience phenotypic alterations with age; the frequency of naïve CD4 and CD8 T cell populations in peripheral blood and in the terminal ileum are lower for older adults (29, 65, 66). This is often accompanied by an increase in T memory cells (66). In fact, we found that naïve aged mice harbored high levels of CD44+ CD4 and CD8 T cells in the spleen and PP compared to adult mice, signifying a greater population of antigen-experienced T cells present prior to vaccination. A proposed explanation for weaker vaccine responses in older individuals is that the naïve T cell pool is reduced and limits primary vaccine responses to novel antigens (20, 67). Altogether, the high bacterial loads observed in aged mice post challenge may be due in part to their inferior T cell responses to CVD 1926.

We observed that CVD 1926 successfully induced antigen-specific fecal IgA responses and functional T cells from the PPs in adult mice, which are characteristic of an effective mucosal immune response. The effect of age on tissue-specific vaccine responses is becoming increasingly appreciated (68). We found that aged mice produced lower antigen-specific IgA levels and weaker PP-derived T cell responses, which are indicative of an impaired mucosal immunity. Consistent with our findings, adults ≥60 years of age that received the live oral vaccine, *S*. Typhi Ty21a also showed lower intestinal T cell cytokine responses compared to younger counterparts (29). As for cell-extrinsic age-related deficiencies, the spleen and lymph nodes of older adults become fibrotic and lose confined stromal networks of the lymphoid tissue (69, 70). It is unknown whether these microenvironment alterations extend to the lymphoid structure of mucosa (e.g., Peyer’s Patches). If so, this may account for blunted mucosal responses to CVD 1926, similarly to how lymphoid tissue fibrosis was associated with impaired immunological responses to yellow fever vaccination (71). Future studies on oral vaccine responses are warranted to better understand mucosal immunosenescence.

In this study, we assessed the effect of vaccination with a live oral *S*. Typhimurium vaccine on several immunological parameters in aged versus adult mice. In addition to immunological changes, other factors could potentially contribute to reduced immunogenicity and efficacy observed in aged mice. For example, increased gut permeability and shifts in intestinal microbiota composition have been shown to be associated with advanced age and may negatively impact vaccine responses (72).

Previous live attenuated *S*. Typhimurium strains that were immunogenic in murine models elicited similar vaccine responses in human volunteers; suggesting that preclinical testing of NTS vaccines in mice can translate clinically (73). Although there are some phenotypic differences in B and T cell populations between older humans and aged mice, the antibody and T cell responses to several licensed vaccines in older adults reflects what has been observed in aged murine models (74). Thus, we anticipate that antibody and T cell responses to CVD 1926 in humans will resemble what we observed herein.

Older adults would highly benefit from vaccines targeting enteric illnesses caused by *Salmonella* spp., *Escherichia coli*, *Clostridium* spp., and others. Live-attenuated vaccines given *via* the oral route can elicit immunity at the local level within the gastrointestinal tract as well as systemically and are easy to administer and manufacture. Overall, this work provides the first head-to-head comparison of NTS vaccine responses between adult and aged mice and shows that the live oral CVD 1926 vaccine was poorly immunogenic in aged mice. The findings herein are important from a translational perspective, as these data indicate that our live NTS vaccine candidate CVD 1926 requires further optimization to be sufficiently immunogenic for older adults. Strategies may include the addition of a mucosal adjuvant, use of a heterologous live oral prime followed by a parenteral boost regimen, and/or mutating *Salmonella* immune evasion genes to increase immunogenicity. Our data also highlight the issue of mucosal immunosenescence and suggest that more research is needed to understand the mechanisms underlying poor immune responses to oral vaccines in aged hosts.

## Data availability statement

The original contributions presented in the study are included in the article/**Supplementary Material**. Further inquiries can be directed to the corresponding author.

## Ethics statement

The animal study was reviewed and approved by University of Maryland School of Medicine IACUC.

## Author contributions

JA designed and performed experiments, analyzed data, prepared figures and prepared the original manuscript draft. FT and SB provided scientific input and technical training. MS provided scientific input. ST conceived and designed experiments, acquired funds for the study, and supervised the study. FT, SB, MS and ST reviewed/edited the manuscript. All authors contributed to the article and approved the submitted version.
