# Supplementary material for: Reduced immunogenicity of a live Salmonella enterica serovar Typhimurium vaccine in aged mice
Source: Front Immunol. 2023 May 3;14:1190339. doi: 10.3389/fimmu.2023.1190339 (PMC10188964; doi:10.3389/fimmu.2023.1190339)
Supplement: Supplementary file 1 [file DataSheet_1.pdf]

Supplementary Material

Reduced immunogenicity of a live *Salmonella enterica* serovar Typhimurium vaccine in aged mice

Jessica C. Allen, Franklin R. Toapanta, Scott M. Baliban, Marcelo B. Sztein, Sharon M. Tennant*

*** Correspondence:** Sharon M. Tennant: stennant@som.umaryland.edu

1 Supplementary Tables

Table S1. Flow cytometry panel for T-CMI assessment.

Marker	Conjugation	Clone	Source
Extracellular/Surface stain			
CD45	FITC	30-F11	Biolegend
CD3	Brilliant Violet 711™	145-2C11	BD Biosciences
CD4	Alexa Fluor® 700	RM4-5	BD Biosciences
CD8	PE/Dazzle™ 594	53-6.7	Biolegend
CD44	Brilliant Violet 650™	IM7	Biolegend
CD19	Biotin	RA3-6B2	Biolegend
F4/80	Biotin	BM8	Biolegend
Streptavidin	Pacific Orange™		Invitrogen
Intracellular Stain			
IFN-γ	V450	XMG.1	BD Biosciences
TNF-α	APC	MP6-XT22	BD Biosciences
IL-2	Brilliant Violet 785™	JES6-5H4	Biolegend

Table S2. Survival and vaccine efficacy in immunized BALB/c mice

Age of mice	No. of surviving animals/ no. of total animals		% Vaccine efficacy	*p*-value (Fisher's exact test)
	PBS	CVD 1926		
Adult	0/15	9/14[a]	64	0.0007
Aged	0/12	4/12	33	0.093

[a], 15 adult mice were immunized with CVD 1926 but one mouse died following the first immunization, most likely due to administration error. This mouse was removed from the analysis.

2 Supplementary Figures

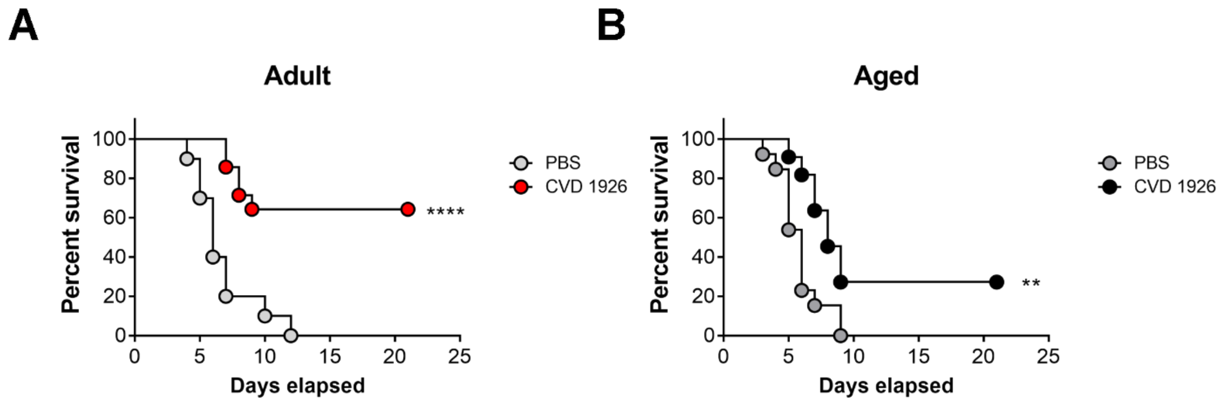

Figure S1. Survival curves of CVD 1926-immunized adult and aged BALB/c mice after challenge with wild-type *S*. Typhimurium. Survival of A) adult (PBS: n=15; CVD 1926: n=14) and B) aged (n=12 for PBS and CVD 1926) BALB/c mice immunized with either PBS or CVD 1926, then subsequently challenged perorally with 100 x LD_{50} (3 x 10^6 CFU) of *S*. Typhimurium I77 (**, $p \leq 0.01$; ****, $p \leq 0.0001$, by log-rank analysis).

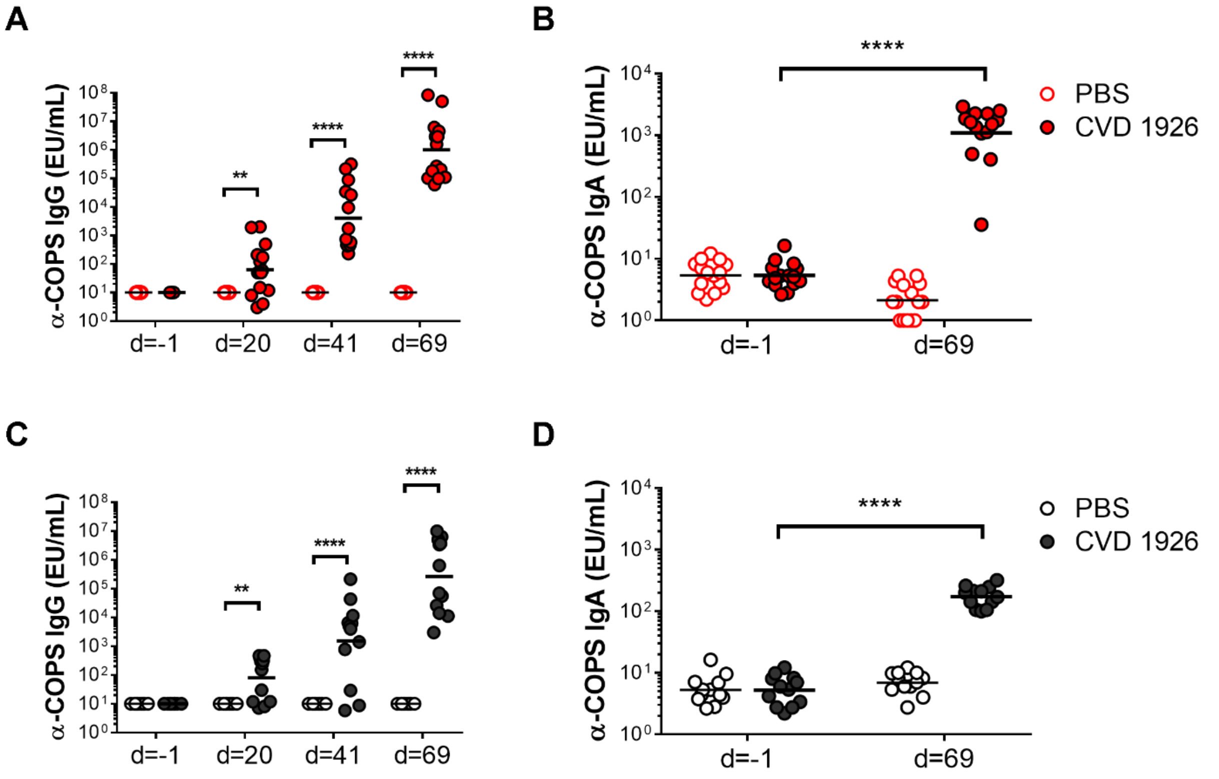

Figure S2. Serum IgG and fecal IgA responses from adult (A and B) and aged (C and D) BALB/c mice immunized with CVD 1926. Anti-COPS serum IgG titers 1 day prior to immunization and challenge (days -1, 20, 41, and 69) in A) adult and C) aged mice. Fecal IgA responses at baseline and 1 day prior to challenge (days -1 and 69, respectively) in B) adult and D) aged mice. Geometric mean titer represented by bar (**, $p \leq 0.01$, ****, $p < 0.0001$ by Mann-Whitney).

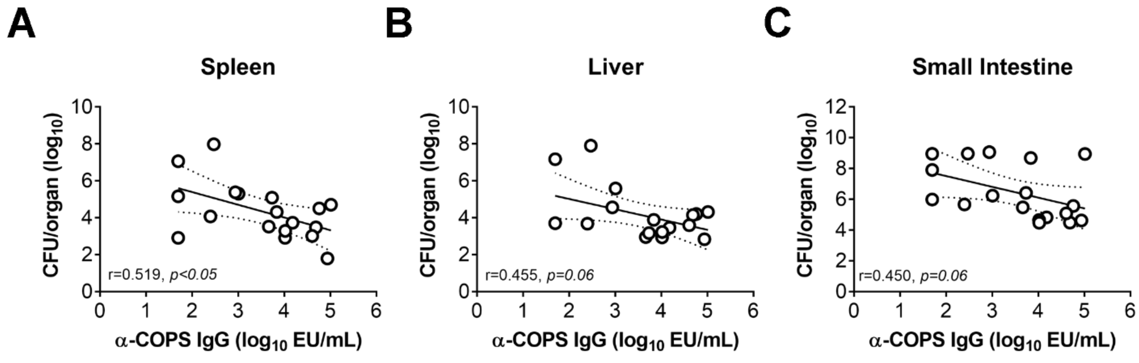

Figure S3. Relationship between anti-COPS serum IgG titers and bacterial burden level in adult and aged mice. Correlation between anti-COPS serum IgG titers and bacterial burden in A) spleen, B) liver and C) small intestine. Significant correlation determined using a Spearman coefficient. *p*-values ≤ 0.05 were considered to be significant.

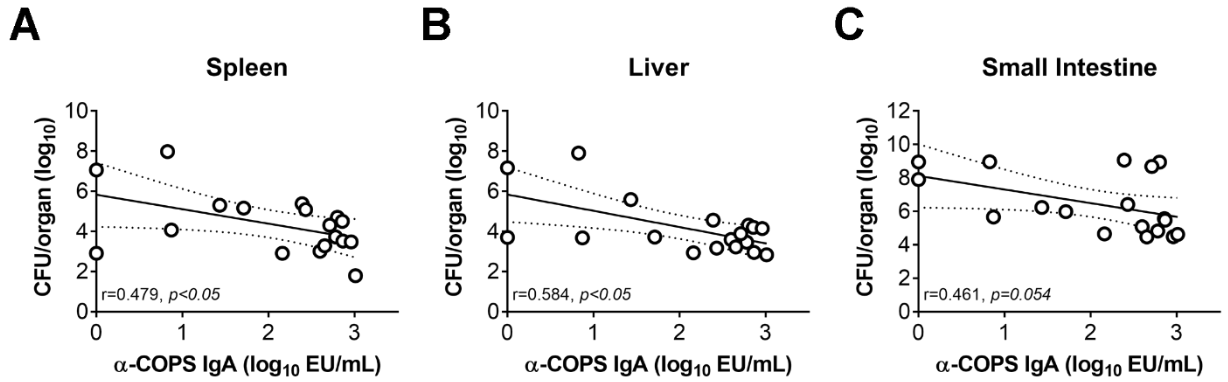

Figure S4. Relationship between anti-COPS fecal IgA titers and bacterial burden level in adult and aged mice. Correlation between anti-COPS fecal IgA titers and bacterial burden in A) spleen, B) liver and C) small intestine. Significant correlation determined using a Spearman coefficient. *p*-values ≤ 0.05 were considered to be significant.

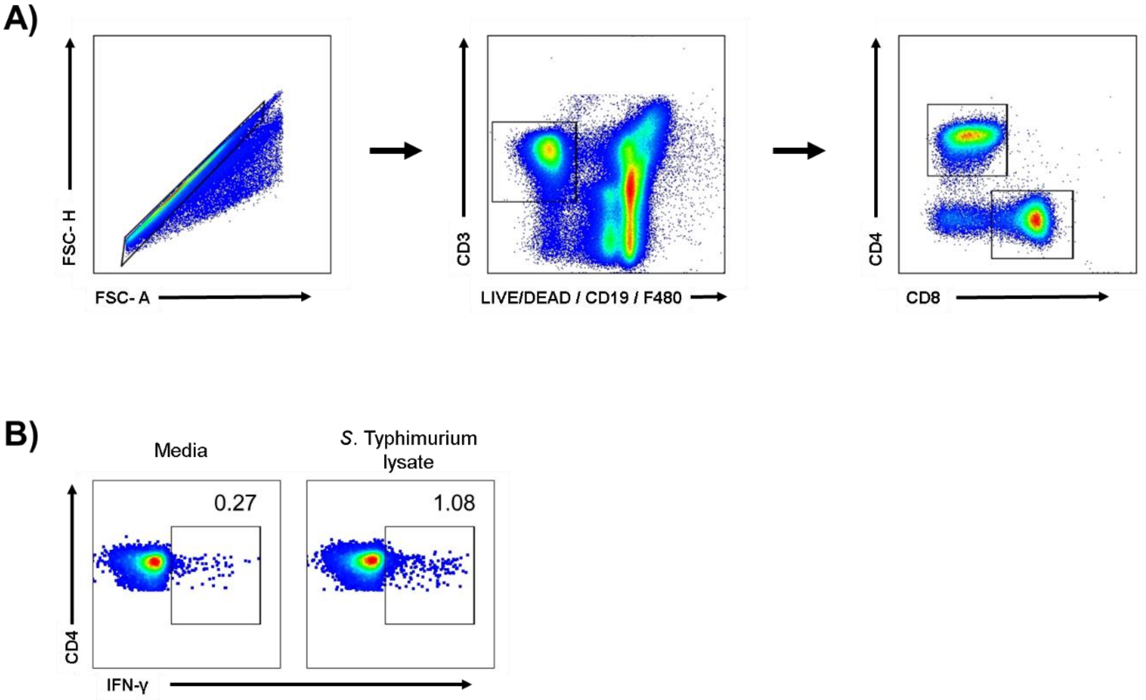

Figure S5. Representative flow cytometry analysis to assess T-CMI. A) Using FSC-H and FSC-A, spleen and PP cells are gated on singlets, followed by CD3+ cells. The live CD3+ population is further separated by CD4 and CD8 surface markers. Once the CD4 and CD8 T cell compartments are defined, B) cytokine production is assessed following *ex vivo* stimulation with *S*. Typhimurium I77 lysate and media alone. Data in panels A and B are from a vaccinated adult mouse.

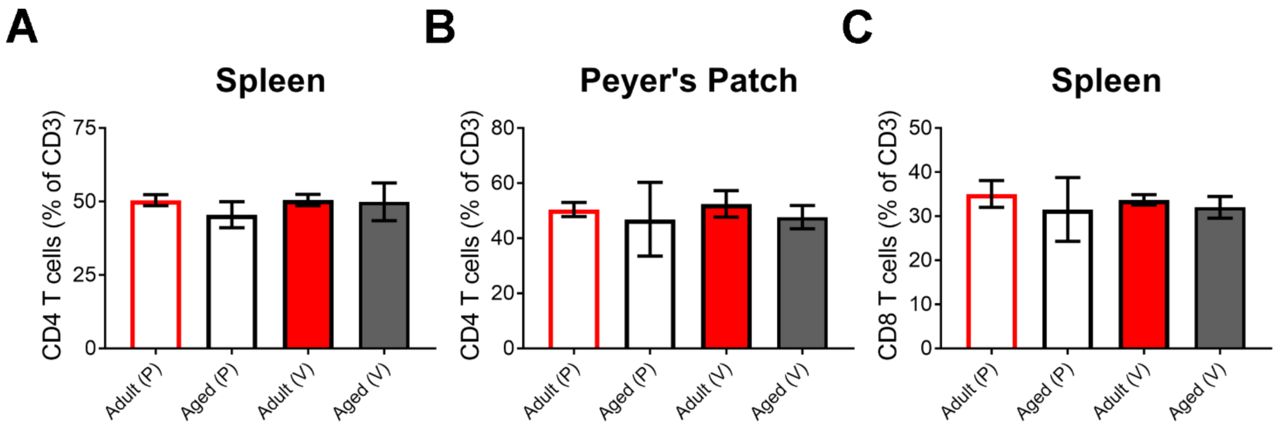

Figure S6. CD4 and CD8 T cell frequencies within the spleen and Peyer's Patches of adult and aged mice. Using multiparameter flow cytometry, the percentage of CD4 T (of CD3+ cells) cells in the A) spleen and B) Peyer's Patches and C) CD8 T cells (of CD3+ cells) within the spleen of adult and aged C57BL/6 mice was measured. Mice receiving placebo (P; PBS) and vaccine (V; CVD 1926) were assessed. Samples collected 14 days following 2 doses of CVD 1926. Data were analyzed by one-way ANOVA.

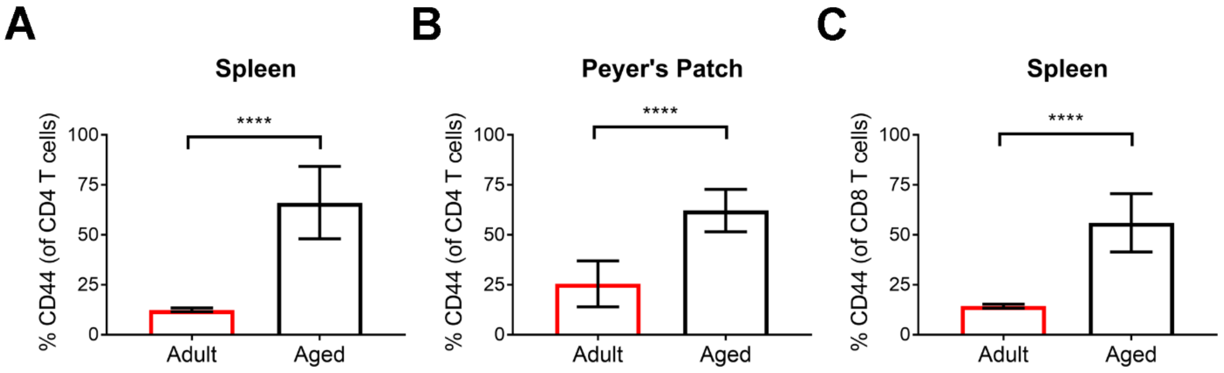

Figure S7. Assessment of memory CD4 and CD8 T cells in the spleen and Peyer's Patches of adult and aged mice. Using multiparameter flow cytometry, the percentage of CD44+ CD4 T cells in the A) spleen and B) Peyer's Patches and C) CD8 T cells in the spleen were measured in C57BL/6 mice (****, $p \leq 0.0001$ by Mann-Whitney).
